# Emerging Strategies in the Diagnosis and Treatment of Pleural Mesothelioma: An Overview

**DOI:** 10.1111/1759-7714.70259

**Published:** 2026-04-23

**Authors:** Raffaella Pagliaro, Beatrice Leonardi, Angela Schiattarella, Grazia Bergameo, Carmine Picone, Adolfo Gallipoli D'Errico, Filippo Scialò, Fabio Perrotta, Maria Luisa De Rimini, Alfonso Fiorelli, Andrea Bianco

**Affiliations:** ^1^ Department of Translational Medical Sciences University of Campania Luigi Vanvitelli Naples Italy; ^2^ Unit of Respiratory Medicine “Luigi Vanvitelli” A.O. Dei Colli, Monaldi Hospital Naples Italy; ^3^ Department of Thoracic Surgery Policlinico Vanvitelli‐University of Campania Naples Italy; ^4^ Division of Radiology Istituto Nazionale Tumori IRCCS Fondazione Pascale‐IRCCS di Napoli. Napoli Italy; ^5^ Department of Medicine and Health Science Vincenzo Tiberio University of Molise Campobasso Italy; ^6^ Lega Italiana Per la Lotta Contro i Tumori Rome Italy; ^7^ Department of Molecular Medicine and Medical Biotechnologies University of Naples Federico II Naples Italy; ^8^ CEINGE‐Biotechnologies Advances Scarl Naples Italy; ^9^ Nuclear Medicine Unit AORN Ospedali Dei Colli Naples Italy

**Keywords:** asbestos, biomarkers, chemotherapy, clinical diagnosis, immunotherapy, pleural mesothelioma (PM), radiotherapy, surgery

## Abstract

Pleural mesothelioma (PM) is a rare and aggressive cancer arising from pleural mesothelial cells with a strong association to asbestos exposure. Among the diagnostic strategies available are noninvasive techniques including thoracic ultrasound (TUS), computed tomography (CT) scans, positron emission tomography (PET‐CT), and invasive procedures such as thoracoscopy and pleural biopsy. Accurate identification of the histological subtype is critical for tailoring treatment strategies. The standard treatment for unresectable PM has traditionally been chemotherapy, particularly platinum and pemetrexed. However, recent advances in translational clinical research, including immune checkpoint inhibitors (ICIs), are changing the therapeutic landscape, offering new opportunities for personalized treatment. The recent FDA approval of nivolumab and ipilimumab combination therapy as a first‐line treatment has significantly improved outcomes, especially for nonepithelioid subtypes. Ongoing studies are exploring additional immune‐targeted therapies such as VISTA, LAG‐3, and dendritic cell‐based therapies. Early detection, refined biomarker identification, and a deeper understanding of the tumor microenvironment remain essential to improving PM prognosis and patient survival. This review provides a comprehensive exploration of the epidemiology, etiology, clinical manifestations, diagnostic approaches (including immunohistochemical and molecular markers), staging, and current treatment strategies for PM.

## Graphical abstract

This review examines pleural mesothelioma, from risk factors and pathogenesis to diagnostic approaches and histological classification. We discuss current treatment strategies including chemotherapy and FDA‐approved immunotherapy, alongside emerging therapies such as VISTA, LAG‐3 inhibitors, and dendritic cell‐based approaches for personalized treatment.

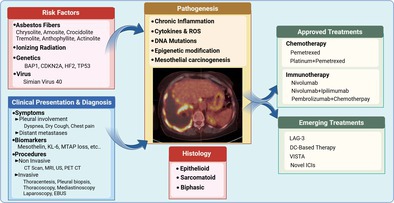

## 1 Introduction

Pleural mesothelioma (PM) is a rare type of cancer originating from pleural mesothelial cells and associated with asbestos exposure. A long latency period ranging from 10 to 50 years between exposure and tumor development is generally observed. Epidemiological studies show that latency is influenced by multiple factors, including the intensity and duration of exposure, occupation, sex, and age at first exposure [1]. Heavier or prolonged occupational exposure is typically associated with shorter latency periods, whereas lighter, indirect, or domestic exposure has been linked to substantially longer latency, with median values exceeding 40 years and upper limits extending beyond 50 years in some populations [2, 3, 4]. The clinical manifestations of PM, including progressive dyspnea, chest pain, and weight loss, are often nonspecific and develop gradually, leading to delayed diagnosis [5].

In 2020, 30 870 mesothelioma cases were reported globally, with an age‐standardized rate (ASR) of 0.30 per 100 000 persons. The highest ASR is found in Northern Europe (1.4), Australia and New Zealand (1.3), Western Europe (0.79), and Southern Europe (0.70) [6].

The exposition to nuclear radiation has also been recognized as a risk factor for the development of PM. Radiation‐associated mesothelioma has been reported following exposure to ionizing radiation from medical treatments such as external beam radiation therapy, diagnostic agents including Thorotrast, and occupational exposure in the nuclear industry. Although mesothelial tissue is not considered highly radiosensitive, epidemiological evidence suggests a modest but measurable increase in mesothelioma risk after both high‐dose short‐term and low‐dose long‐term radiation exposure. Patients who develop PM following radiation exposure tend to be diagnosed at a younger age and may experience longer overall survival compared with asbestos‐related cases, potentially reflecting differences in exposure patterns, latency, comorbidities, and tumor biology [7]. Age at first asbestos exposure is another important determinant of mesothelioma latency. Epidemiological studies have shown that latency decreases with increasing age at initial exposure, with individuals exposed later in life developing PM after shorter latency periods compared with those exposed at younger ages, who typically experience longer latency intervals before diagnosis. This finding supports the concept that both host‐related factors and timing of carcinogenic exposure influence disease onset [3]. In addition to environmental exposures, genetic susceptibility plays a significant role in mesothelioma development. Germline and somatic alterations in tumor suppressor genes can increase individual vulnerability to asbestos‐ and radiation‐induced carcinogenesis. In particular, mutations in the BRCA1‐associated protein 1 (BAP1) gene are strongly associated with earlier disease onset and increased mesothelioma risk, even after relatively low‐level asbestos exposure [8]. Other recurrently altered genes in PM include CDKN2A, NF2, LATS2, and TP53, which are involved in cell‐cycle regulation, DNA damage response, and Hippo signaling. These genetic alterations do not independently cause mesothelioma but may accelerate tumor development and influence disease aggressiveness in exposed individuals [8, 9]. Other genetic variants, including those in DNA repair genes such as PALB2, BRCA1/2, and the cyclin‐dependent kinase inhibitor 2A (CDKN2A), have also been associated with an accelerated development of PM [10].

Most patients are diagnosed with PM at an advanced stage, resulting in a poor prognosis with a median survival time of around 12–14 months [11]. The recent FDA approval of the combination of nivolumab (an anti‐PD‐1 inhibitor) and ipilimumab (a CTLA‐4 inhibitor) in patients with unresectable advanced or metastatic PM offers a new perspective in terms of life expectancy [12].

This review aims to provide an overview of the pathogenesis, clinical presentation, diagnostic approaches, and emerging therapeutic strategies for PM, highlighting the need for early detection and novel treatment options to improve patient survival and quality of life (QoL).

### 1.1 Pathogenesis of PM

PM is primarily related to asbestos exposure. Asbestos refers to a group of six varieties of fibrous minerals like chrysotile (serpentine), amosite, and actinolite (amphibole) as well as anthophyllite, crocidolite, and tremolite [8]. The six main types of asbestos fibers can be categorized into two groups based on their structure: Serpentine (curly fibers: chrysotile) and Amphibole (straight, needle‐like fibers: amosite, crocidolite, tremolite, anthophyllite, and actinolite). All types are carcinogenic, but amphibole fibers are generally considered more hazardous due to their rigid, rod‐like structure, which allows them to penetrate and persist in lung tissue more easily. The relative carcinogenic potential of each type is summarized in Table 1. Among these, crocidolite is recognized as the most potent carcinogen [13].

**TABLE 1:** Classification of asbestos types by mineral group, fiber morphology and relative carcinogenic potential highlighting differences between serpentine and amphibole asbestos and their associated health risks.

Asbestos type	Group	Fiber shape	Carcinogenic potential	Notes
Chrysotile	Serpentine	Curly, flexible	Moderate	Most common in commercial use
Amosite	Amphibole	Straight, needle‐like	High	Strongly associated with mesothelioma
Crocidolite	Amphibole	Straight, needle‐like	Very High	Considered most potent carcinogen
Tremolite	Amphibole	Straight, needle‐like	High	Often contaminant in chrysotile
Anthophyllite	Amphibole	Straight, needle‐like	High	Less common
Actinolite	Amphibole	Straight, needle‐like	High	Less common

Due to their characteristics (longer than 8.0 and 0.25 μm thick), the fibers can remain in the pleura for prolonged periods, triggering various pathogenic processes. The initial response is local inflammation, which causes repeated tissue injury and repair, leading to scarring or potential progression to malignancy [2, 13, 14, 15]. There is a strong link between the local and systemic inflammatory responses, and the patient's prognosis [13].

Indeed, following local inflammation, an intense and persistent systemic inflammatory response is triggered, characterized by the migration of white blood cells and the secretion of cytokines, which induce malignant changes in mesothelial cells [13, 16]. Tumor‐associated macrophages (TAMs), regulatory lymphocytes (Treg), and myeloid‐derived suppressor cells (MDSCs) are recruited by malignant cells [17]. These cells play a key role in promoting the formation of new blood vessels, remodeling the extracellular matrix, evading immune surveillance, and facilitating tumor progression [14, 17]. Asbestos, in particular crocidolite, causes the accumulation of macrophages in the pleura and lung, leading to the subsequent production of TNFA [13, 18]. Furthermore, this fiber also stimulates mast cells to release TNFA and produce the TNFA receptor, TNF‐R1, causing subsequent paracrine and autocrine reactions [18]. TNFA, in turn, triggers the nuclear factor kB (NF‐kB) pathway, resulting in DNA damage and subsequent development of PM [18]. DNA damage can occur in two ways: (I) by triggering the production of oxygen free radicals responsible for intracellular DNA damage and disruption of repair mechanisms [19] and (II) by penetrating mesothelial cells, interfering with mitosis and apoptosis and thus generating mutations in DNA resulting in alterations in chromosome structure [14, 17]. Indeed, the loss of one copy of chromosome 22 is often observed in PM patients [20]. Other chromosomal abnormalities identified include deletions in chromosome arms 3p, 1p, 6q, and 9p [20]. Furthermore, it is documented that asbestos is also able to interfere with early response proto‐oncogenes which, in turn, promote abnormal cell proliferation through the mitogen‐activated protein kinase (MAP) and extracellular signal‐regulated kinase (ERK) 1 and 2 pathways [14]. Additional environmental risk factors in the pathogenesis of PM have been recognized, as well as microbiome [21, 22].

**FIGURE 1:**
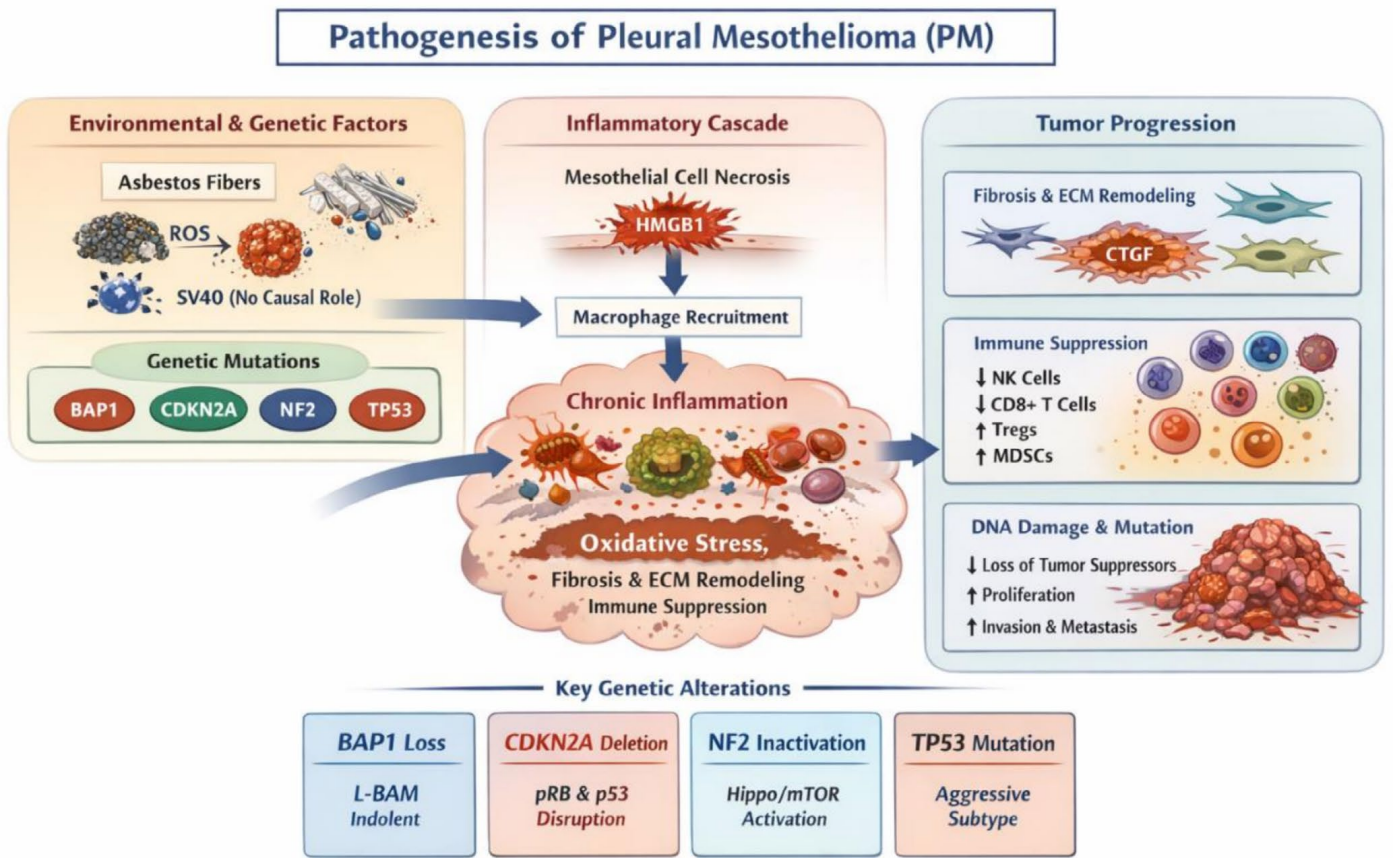
Schematic representation of PM pathogenesis. Environmental, genetic, and biological factors interact to drive PM development. Environmental triggers include chronic asbestos exposure, which induces reactive oxygen species (ROS), interferes with mitosis, and activates macrophages. These events lead to mesothelial cell necrosis and the release of HMGB1, recruiting macrophages that secrete pro‐inflammatory cytokines (IL‐1β, TNF‐α, IL‐6, IL‐8, TGF‐β, VEGF), establishing a chronic inflammatory microenvironment. This milieu promotes oxidative stress, fibrosis, extracellular matrix (ECM) remodeling, angiogenesis, and immune suppression, facilitating tumor progression. Genetic alterations in tumor suppressor genes (BAP1, CDKN2A, NF2, TP53) further exacerbate malignant transformation and influence disease aggressiveness and subtype. BAP1 loss is associated with low‐grade, indolent mesothelioma (L‐BAM), CDKN2A deletion disrupts pRB/p53‐mediated checkpoints, NF2 inactivation activates Hippo/mTOR signaling, and TP53 mutations drive aggressive phenotypes. Collectively, these processes lead to mesothelial malignant transformation, tumor invasiveness, and resistance to therapy. SV40 is depicted for historical context but has no causal role in PM pathogenesis.

Previous exposure to ionizing radiation, such as mantle radiation for Hodgkin's lymphoma, or occupational exposure to other mineral fibers such as erionite and synthetic materials (in the case of ceramics, railway repairs, auto parts manufacturers, mining, etc.) [14, 15, 18]. In addition, Regis et al. described simian virus 40 (SV40) as a potential cofactor in the pathogenesis of PM, although its role is controversial [23] Moreover, cases of PM inherited in an autosomal dominant form also have been described in the literature [24]. Schematic representation of PM pathogenesis is shown in Figure 1.

### 1.2 Clinical Presentation

Many patients with PM present nonspecific symptoms that typically occur in the sixth or seventh decade of life, several years after asbestos exposure. Common symptoms include progressive dyspnea, dry cough, and chest pain which may be linked to pleural effusion or volume reduction associated with the disease. Chest pain is often pleuritic and is usually caused by the tumor's locally invasive nature, leading to bone erosion or nerve compression. As the disease progresses, patients may experience fatigue, anorexia, weight loss, sweats, and general malaise. Extrapulmonary restriction with ventilatory impairment may be present, typically showing neither obstructive nor restrictive patterns. This is reflected in a reduction in forced expiratory volume in 1 s (FEV1) and forced vital capacity (FVC), while the FEV1/FVC ratio remains normal. Total lung capacity and gas transfer are decreased, along with an increased diffusion constant, as seen in cases of diffuse pleural thickening [25].

Although PM is predominantly characterized by malignant local infiltration [26], distant metastases are reported more frequently than previously implied. Bone metastases (BoM) have been described in up to 3% of MPM patients [5, 6], and brain metastases have also been documented, emphasizing the need to recognize extrathoracic dissemination in selected patients [27, 28]. Histological subtype influences metastatic patterns and prognosis: sarcomatoid and biphasic subtypes are associated with more aggressive behavior and shorter survival [10, 14, 17]. BoM is more likely in patients with nonepithelial histology (OR 2.189, 95% CI 1.179–4.065, *p* = 0.013), underscoring the importance of early identification and monitoring. The potential association between BoM and other distant metastatic sites, such as the liver, lungs, or brain, remains an area for further investigation. Patients at high risk may benefit from periodic imaging, including FDG‐PET or brain MRI, even if asymptomatic. Extended follow‐up studies are essential to determine the true incidence, timing, and prognostic impact of distant metastases in PM, particularly in the context of evolving therapies such as immunotherapy.

In conclusion, while PM is largely a locally invasive thoracic malignancy, distant metastases—including bone and brain—can occur and significantly affect survival and QoL. Clinicians should carefully screen patients with high‐risk features to enable early detection and appropriate management.

### 1.3 Diagnostic Procedures

The accurate diagnosis and staging of PM requires a combination of imaging exams and invasive procedures. These methods can be categorized into noninvasive and invasive techniques [29]. Noninvasive techniques include CT scans, magnetic resonance imaging (MRI), ultrasound, positron emission tomography/computed tomography (PET‐CT) and pulmonary function tests [30, 31]. Invasive examinations include pleural biopsy (guided by computed tomography [CT] or ultrasound), thoracoscopy, mediastinoscopy, and in some cases, laparoscopy [32, 33]. Endobronchial ultrasound‐guided fine needle aspiration may be employed for mediastinal staging.

#### 1.3.1 CT‐Scan

Contrast‐enhanced chest CT is an essential step of PM diagnosis, as it can highlight pleural features indicative of malignancy [34]. These features include nodular or circumferential pleural thickening, or mediastinal and parietal pleura thickening greater than 1 cm [35]. There is ongoing debate regarding whether pleural fluid drainage should precede CT scanning, as conducting the scan before drainage improves the visualization of the pleura. However, this approach may not provide accurate details of potential lung parenchymal lesions [36]. The Ninth Edition TNM for PM updates staging by introducing quantitative size criteria (pleural thickness) for the T category, alongside invasion, while there are no changes to the N and M categories. Particular interest is directed toward intrascissural involvement; the key metric introduced is Fmax (maximum fissure thickness) [37]. Contrast CT scan is considered the gold standard to assess this parameter: a measured *F*_max_ > 5 mm serves as an independent predictor of poor prognosis, showing survival rates similar to the T2 category.

#### 1.3.2 FDG‐PET Scan

**FIGURE 2:**
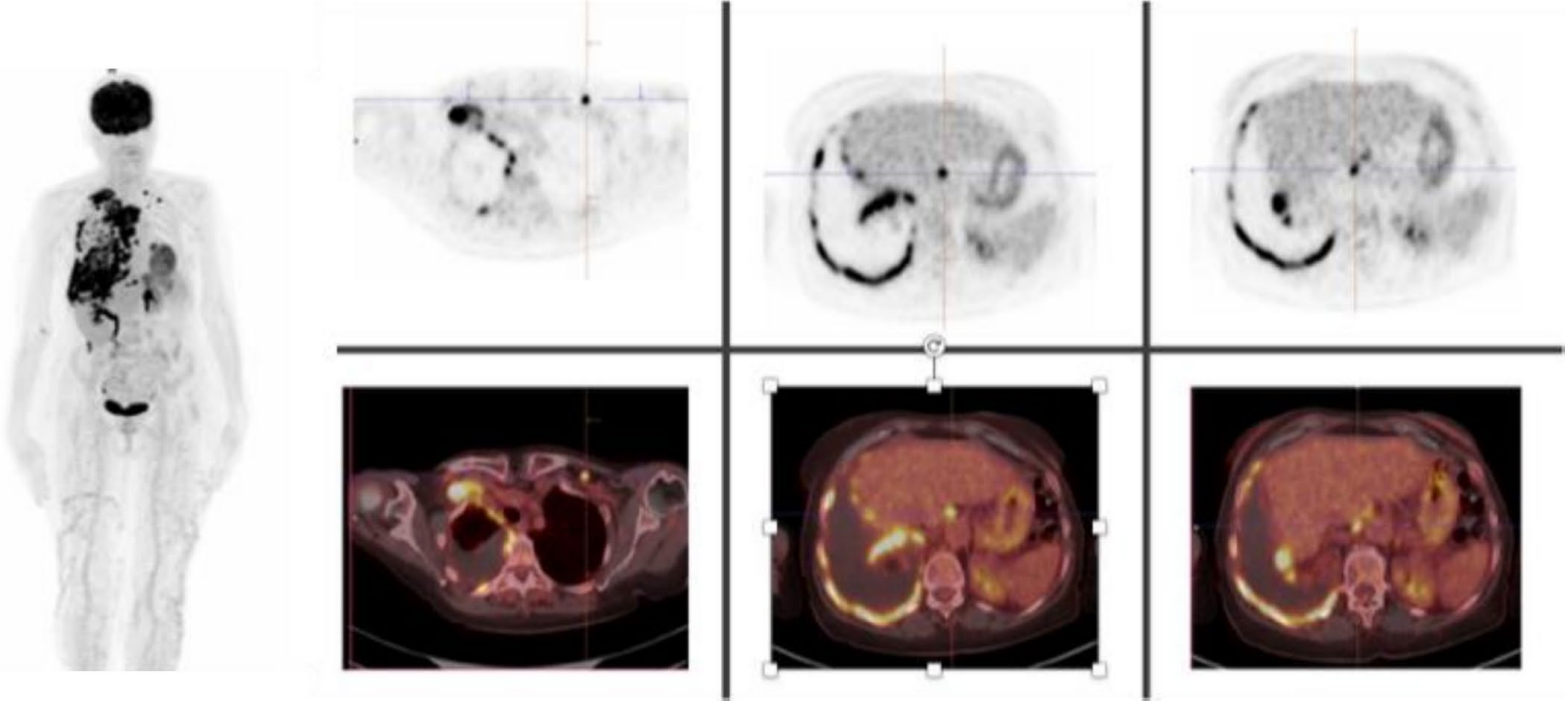
18F‐FDG PET/CT images showing multiple areas of pathological uptake. The maximum intensity projection (left) highlights widespread hypermetabolic lesions. Axial PET images (top row) and fused PET/CT images (bottom row) demonstrate increased FDG uptake in various sites.

**FIGURE 3:**
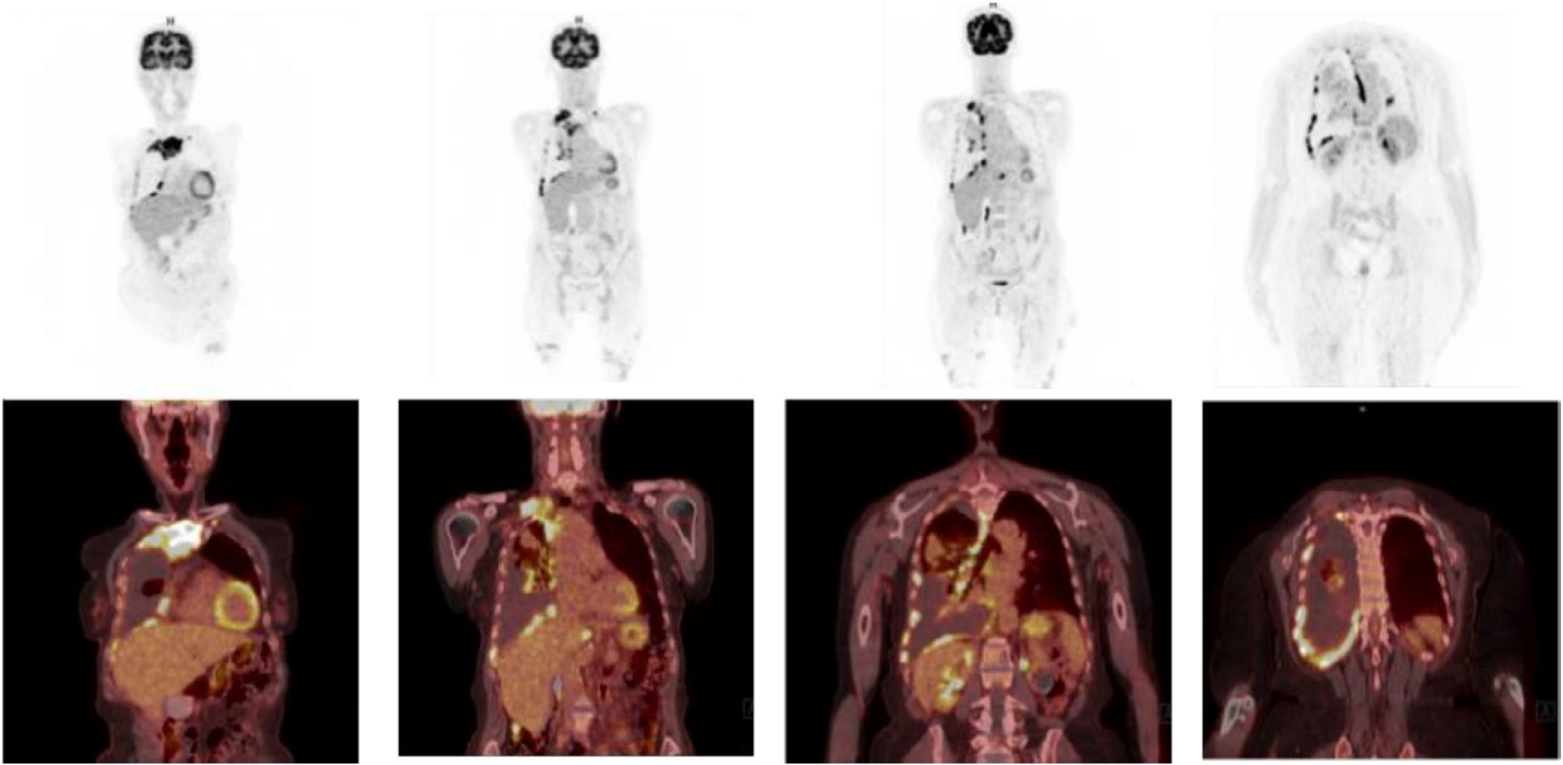
18F‐FDG PET/CT images showing multiple areas of pathological uptake. Maximum intensity projection images (top row) highlight widespread hypermetabolic lesions. Corresponding fused PET/CT images (bottom row) demonstrate intense FDG uptake in multiple regions, including thoracic and abdominal sites, indicating metabolically active disease.

FDG‐PET scan 18 F‐fluorodeoxyglucose—positron emission tomography (PET‐CT) is a functional imaging technique that concurrently provides anatomical CT reconstructions and metabolic activity of the examined district [38, 39]. A maximum SUV threshold of 2 reliably differentiates PM from benign disease, given that PM demonstrates higher SUV values [40]. The accuracy of PET‐CT can be compromised by factors such as pleural inflammation/infection or previous talc pleurodesis, which may lead to false positives [41]. Additionally, PET‐CT scan is a valuable tool for identifying specific biopsy targets in cases where medical thoracoscopy is not feasible or has failed to provide a definitive diagnosis through tissue sampling [36]. The importance of the role of PET/CT in the management of PM is illustrated by two examples, as shown in Figures 2 and 3.

#### 1.3.3 The Role of Thoracic Ultrasound (TUS)

TUS has improved the diagnosis and management of pleural diseases [42]. According to the analysis of Qureshi et al., TUS reported a sensitivity of 73% and a specificity of 100% in distinguishing between malignant and benign pleural conditions [43]. However, findings such as pleural thickening greater than 1 cm, pleural nodularity, and diaphragmatic thickening exceeding 7 mm are indicative of malignancy [44]. Its role is central in managing pleural effusions and serves as a guide for interventional procedures in patients not suitable for thoracoscopy [33] and as a secondary “on‐the‐table” option if thoracoscopy fails, as reported in ERS/ESTS/EACTS/ESTRO guidelines [45]. Furthermore, the utility and accuracy of TUS‐guided percutaneous needle biopsy have been evaluated, demonstrating its effectiveness in diagnosing PM, especially in patients who are not candidates for thoracoscopy [46]. TUS is currently recommended to guide most pleural procedures, reducing the risk of complications such as accidental organ puncture, pneumothorax, and bleeding from intercostal and internal mammary arteries [36].

### 1.4 Invasive Diagnostic Procedures

#### 1.4.1 Thoracentesis

Therapeutic thoracentesis should be performed when patients present with symptomatic pleural effusions. Pleural fluid cytology and immunocytochemistry are often inconclusive for the diagnosis of PM, with a sensitivity ranging between 30% and 75% and a specificity between 80% and 99% [47].

Although less than one‐third of PM can be diagnosed accurately on pleural fluid cytology, thoracentesis is a safe and reliable initial intervention that can transiently alleviate dyspnea and chest discomfort [48]. However, thoracentesis has a low sensitivity in detecting the sarcomatoid and biphasic subtypes of PM, although it can be useful in diagnosing the epithelioid subtype [43].

#### 1.4.2 Thoracoscopy/Open Pleural Biopsy

Large biopsies and proper immunohistochemistry (IHC) are essential for distinguishing mesothelioma from other tumors and determining its histological subtype [33]. Pleural biopsies are obtained through medical thoracoscopy or video‐assisted thoracoscopic surgery [49]. Multiple large biopsies that include subpleural tissue for the histological assessment of invasion can be obtained.

In patients with advanced disease, factors such as local invasion, pleural thickening, absence of pleural effusion, and volume loss can make thoracoscopic biopsy challenging. If thoracoscopy is not feasible, an open pleural biopsy should be considered, using the smallest incision possible (typically 6 cm or less). The diagnostic yield of thoracoscopy for PM is over 95% [50].

### 1.5 New Diagnostic Procedures

#### 1.5.1 Artificial Intelligence (AI) for PM

In recent years, AI has been implemented in the diagnosis of various conditions. The application of AI for PM patients is promising with respect to improving diagnostic accuracy and identifying prognostic factors for this disease. Latif et al. used databases of PM patients to identify the risk factors for this neoplasm as early as possible [51]. The authors of this research believe that AI and data analysis of PM patients could be useful in early diagnosis but also in the management of comorbidities of patients with this disease. Other studies focused on the early identification of individuals at risk of developing PM and of patients with a worse prognosis [52, 53]. Various machine‐learning algorithms have been employed to facilitate the early identification of PM patients and to support both diagnostic and prognostic processes [54]. In particular, techniques such as AdaBoost and other advanced AI frameworks offer noninvasive and cost‐effective solutions for prognostic evaluation. Furthermore, progress in AI models, including large language models (LLMs), has enhanced the accuracy of differential diagnosis by enabling clearer disease classification [55]. Finally, the development of SpindleMesoNET—a specialized neural network—demonstrates AI's potential to effectively distinguish between malignant and benign mesothelial proliferations [56]. These findings are summarized in Table 2.

**TABLE 2:** Risk factors for mesothelioma development and characteristics of patients at high risk for poor clinical outcomes.

Category	Factor	Notes/evidence
Environmental risk factors	Asbestos exposure	Chronic occupational or environmental exposure; latency 10–50 years; all fiber types carcinogenic, amphibole fibers (crocidolite, amosite, tremolite, anthophyllite, actinolite) most potent
	Ionizing radiation	External beam radiation, Thorotrast, nuclear industry exposure; modest increased risk; younger age at presentation; longer survival in radiation‐associated cases
	Other mineral fibers	Erionite, synthetic fibers (ceramics, railway, mining, auto parts)
	SV40	Previously suggested cofactor, current evidence does not support causal role
Genetic susceptibility	BAP1	Germline or somatic mutations; early‐onset, slower progression; low‐grade BAP1‐associated mesothelioma (L‐BAM) subtype
	CDKN2A	Deletion disrupts pRB/p53 checkpoints; associated with aggressive disease
	NF2	Loss of Merlin protein; activates Hippo/mTOR pathways; promotes proliferation and invasion
	TP53	Functional loss accelerates tumor progression; associated with highly invasive subtypes
Patient characteristics associated with poor outcomes	Age	Older age at diagnosis associated with worse prognosis; younger age may present with radiation‐associated PM
	Histology	Sarcomatoid and biphasic subtypes associated with shorter survival
	Disease stage	Advanced IMIG stage correlates with poor survival and higher risk of metastasis
	Distant metastases	Bone (BoM) and brain metastases; BoM linked with nonepithelial histology; adversely affects survival and quality of life
	Comorbidities/systemic factors	Chronic inflammation, immunosuppression, fibrosis, oxidative stress contribute to poor outcomes

### 1.6 Biomarkers

In the cancer landscape of biomolecular profiling, liquid biopsy is increasingly applied for the early identification of at‐risk subjects, diagnosis, treatment, disease progression, and prognosis [57, 58, 59]. In this context, various markers have been examined to define an optimal, noninvasive biomarker for diagnosing and monitoring PM [60]. Several studies have investigated the role of serum mesothelin as a useful biomarker for monitoring PM; in particular, reductions in serum mesothelin levels after treatment have been linked to positive treatment responses, tumor shrinkage, and improved survival outcomes [61, 62]. Moreover, osteopontin (OPN) was evaluated as an indicator of the duration of asbestos exposure, demonstrating that serum OPN levels correlated with survival at baseline [63]. According to a recent study, Krebs von den Lungen‐6 (KL‐6), may be a potential novel liquid‐based diagnostic and prognostic biomarker for PM. High pleural KL‐6 levels correlate with the epithelioid subtype and, interestingly, predict better overall survival, acting as a favorable prognostic indicator [64]. Furthermore, peripheral blood eosinophils have emerged as a promising biomarker. According to Willems et al. [65], an excess of blood eosinophils (defined as ≥ 220/μL) prior to starting therapy correlates with a worse prognosis. This suggest that high eosinophil levels may promote a pro‐tumor microenvironment, adding a biological layer to our understanding of the disease's progression. Other studies have shown promising results for HMGB1 and CD138, but further validation is required for their diagnostic use [66]. Zhu. et al. evaluated the diagnostic value of DNA and combinations of multiple markers for PM using a meta‐analysis approach. With the comparison of 13 different diagnostic groups, methylthioadenosine (MTAP) gene expression was associated with longer survival in PM patients, suggesting the potential role of MTAP regulation in improving patient outcomes. Furthermore, fibulin 3 was identified as a potential biomarker for early diagnosis of PM, although its specificity is limited. Nevertheless, MTAP, when used in combination with IHC, could enhance diagnostic specificity and provide prognostic insights [67]. Additionally, some research has shown that miRNAs are dysregulated in PM, with these molecules playing a crucial role in the disease's development and progression [68]. Benjamin et al. identified specific miRNA biomarkers that enable differential diagnosis of PM and developed a diagnostic assay based on miRNA expression in tissue [69]. Other biomarkers have shown prognostic utility in individual studies but further validation in large cohort studies is needed.

**FIGURE 4:**
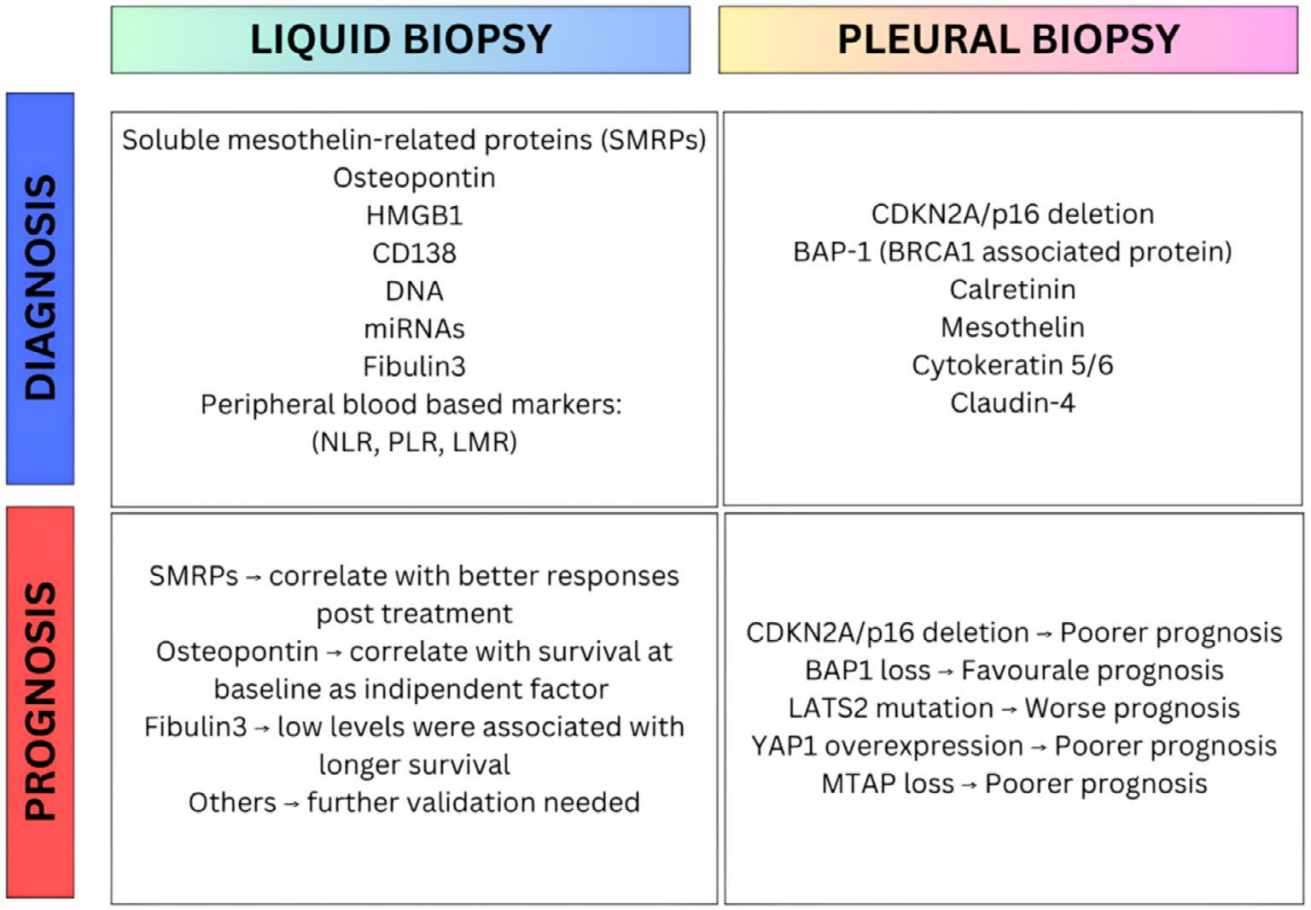
Diagnostic and prognostic biomarkers in PM according to the sample type and clinical application. Liquid biopsy‐based biomarkers include soluble mesothelin‐related proteins (SMRPs), osteopontin, fibulin‐3, HMGB1, CD138, circulating DNA, and microRNAs. SMRPs are associated with better posttreatment response, while osteopontin represents an independent baseline prognostic factor for survival. Low circulating levels of fibulin‐3 are associated with longer survival, whereas other circulating biomarkers require further clinical validation. Peripheral blood–based inflammatory markers, including neutrophil‐to‐lymphocyte ratio (NLR), platelet‐to‐lymphocyte ratio (PLR), and lymphocyte‐to‐monocyte ratio (LMR), provide additional prognostic information.

However, a pleural biopsy along with an array of IHC markers like p16, BAP1, and claudin‐4, is essential for making a definitive diagnosis [70]. The molecular features of PM have been shown to influence the prognosis and thereby are being proposed for inclusion in histopathological reports. These markers are CDKN2A (p16) deletion (associated with poorer prognosis) [71], BAP1 loss (associated with favorable prognosis), LATS2 mutation (associated with worse prognosis), and YAP1 overexpression [72]. Other genetic biomarkers such as p53, NF2, W1F1, and DNA methylation have been associated with predicting prognosis but have limited clinical relevance [73, 74]. In addition, a recent study demonstrated that BAP1 loss, identified by IHC, predicted better overall survival after the first line of therapy with platinum‐pemetrexed [75]. Figure 4 provides a summary of the roles of key biomarkers.

Pleural biopsy–derived biomarkers include diagnostic markers such as calretinin, mesothelin, cytokeratin 5/6, and claudin‐4, as well as molecular alterations with prognostic relevance. CDKN2A/p16 deletion, frequently co‐deleted with MTAP, is associated with poorer prognosis, whereas BAP1 loss is generally associated with a more favorable outcome. Conversely, LATS2 mutations and YAP1 overexpression correlate with worse prognosis.

### 1.7 Past and New Therapeutic Strategies

The treatment landscape for unresectable PM has evolved over the past two decades. Platinum‐pemetrexed chemotherapy established the first‐line standard of care following the phase III EMPHACIS study which demonstrated superior median OS compared to platinum alone (12.1 vs. 9.3 months), leading to regulatory approval in 2004 [76]. Multikinase inhibitors targeting multiple aspects of angiogenesis are used in clinical trials in combination with chemotherapy but none of them have received approval for clinical practice [77]. In particular, the MAPS Phase 2 trial conducted by a French research group demonstrated that adding bevacizumab to cisplatin and pemetrexed improved OS and QoL compared with chemotherapy alone (median OS 18.8 vs. 16.1 months; HR 0.77). However, the combination was associated with significantly higher toxicity, including increased rates of Grade 3/4 hypertension, proteinuria, and thrombotic events. Despite these positive outcomes, regulatory approval for this regimen was not pursued in either Europe or the United States. Moreover, unlike nonsmall cell lung cancer with nonsquamous histology, where maintenance pemetrexed improves OS, there is no evidence supporting pemetrexed maintenance in PM. The Dutch NVALT19 trial showed that switch maintenance with gemcitabine after platinum–pemetrexed chemotherapy improved PFS compared with best supportive care (6.2 vs. 3.2 months; HR 0.48), but without an OS benefit and with substantially higher Grade 3/4 adverse events. Ongoing research includes the Phase 2 TALAMESO trial, which is evaluating maintenance talazoparib, a PARP inhibitor, following first‐line chemotherapy in pleural and peritoneal mesothelioma. In general, regarding subsequent therapies, a meta‐analysis suggested that platinum re challenge combined with pemetrexed, or pemetrexed alone, represents one of the most active second‐line treatment options. Single‐agent chemotherapy with vinorelbine or gemcitabine is commonly used in clinical practice; however, no survival benefit has been demonstrated for vinorelbine compared with placebo (VIM randomized trial), nor for vinorelbine or gemcitabine compared with pembrolizumab monotherapy (PROMISE‐meso randomized trial).

In the phase II randomized RAMES trial, the combination of gemcitabine and ramucirumab showed a promising overall survival benefit compared with gemcitabine plus placebo (median OS 13.8 vs. 7.5 months; HR 0.71, 70% CI 0.59–0.85; *p* = 0.028) in patients who had progressed after standard first‐line chemotherapy.

Several studies have evaluated immune checkpoint inhibitors (ICIs) in later lines of treatment, with heterogeneous and overall modest activity. Objective response rates ranged from approximately 10% to over 40%, with highly variable progression‐free and overall survival outcomes.

The Phase III CONFIRM trial is the only randomized study providing evidence in the third‐line setting compared with placebo in ICI‐naive patients, demonstrating a significant improvement in both overall survival (HR 0.72, 95% CI 0.55–0.94) and progression‐free survival. At present, the use of treatments beyond the second line remains largely investigational.

A promising therapy that has changed the cancer treatment scenery is the use of ICIs [78, 79, 80, 81, 82]. However, in PM ICIs have first been investigated in relapsed disease. The phase III PROMISE‐Meso trial did not show a survival advantage for pembrolizumab (PD‐1 inhibitor) compared to standard chemotherapy [83]. However, the phase III CONFIRM trial demonstrated a survival benefit for nivolumab (PD‐1 inhibitor) over placebo in relapsed PM, with overall survival of 10.2 months versus 6.9 months (*p* < 0.01) [84]. Supporting the inclusion of ICIs in PM treatment, MAPS2 was a noncomparative, Randomized Phase 2 trial that evaluated the effectiveness of nivolumab alone versus a combination of nivolumab and ipilimumab (anti‐CTLA‐4 antibody) in patients with relapsed disease. The trial found similar disease control rates between the two regimens, with 44% of patients on nivolumab and 50% of those on the nivolumab‐ipilimumab combination remaining progression‐free at 12 weeks [85].

The Phase III CheckMate 743 study, which involved 605 patients with unresectable PM, compared first‐line platinum‐based CT with a combination of nivolumab plus ipilimumab as first‐line treatment. The median OS was 18.1 months for the IT group compared to 14.1 months for the CT group (HR 0.74, *p* = 0.002) [86]. However, the most significant benefit was observed in nonepithelioid patients (18.1 vs. 8.5 months with chemotherapy). In addition, although the overall response rate (ORR) was similar between two groups (43% vs. 40%), immunotherapy led to more durable responses, with 32% of patients showing ongoing responses at 2 years compared to only 8% in the chemotherapy group. An exploratory analysis also found that a four‐gene inflammatory signature (including PD‐L1, LAG‐3, CD8a, and STAT1) was associated with improved OS in patients treated with immunotherapy (21.8 vs. 16.8 months, HR 0.57), but not in those treated with chemotherapy (11.6 vs. 15.2 months, HR 1.14) [12]. At 3 years, the PFS rates were 14% versus 1% and the OS rates were 17% versus 11% [87]. The results indicated that nivolumab and ipilimumab are the standard of care treatment for unresectable PM and this regimen is approved by FDA; the magnitude of benefit was larger for patients with nonepithelioid versus epithelioid histology [88]. Furthermore, Nivolumab for PM was first approved as a second‐line therapy by PMDA in Japan based on the MERIT trial [89], that showed an objective response rate of 29% and a manageable safety profile. More recently, Higashiyama et al. [90] reinforced the clinical efficacy and safety of nivolumab, emphasizing its active administration for patients with a good Performance Status (PS 0–1), even in later treatment lines. The study proposes that nivolumab administration as maintenance therapy should be considered before PS decreases due to tumor progression in patients treated with first‐line cytotoxic chemotherapy.

In parallel, pembrolizumab combined with platinum‐pemetrexed chemotherapy demonstrated a significant OS benefit compared with chemotherapy alone in the Keynote‐483 trial. The combination achieved a longer median OS (12.3 vs. 8.2 months, HR 0.57 [95% CI 0.36–0.89]) with a manageable safety profile. Although treatment‐related Grade 3–4 adverse events and hospitalizations occurred more frequently in the pembrolizumab arm, long‐term survival outcomes favored the combination regimen [91]. KEYNOTE‐483 confirmed the efficacy of chemoimmunotherapy, whereas CheckMate 743 highlighted the preferential benefit of combination PD‐1/CTLA‐4 blockade in nonepithelioid histologies. In epithelioid PM, OS gains were modest and did not reach statistical significance, although the high ORR with pembrolizumab‐based regimens suggests early cytoreductive activity and symptomatic improvement [92]. These data support a histology‐driven algorithm, favoring nivolumab plus ipilimumab in nonepithelioid PM, immunologically “hot” tumors for durable control, while chemoimmunotherapy remains appropriate for epithelioid or frail patients requiring rapid tumor burden reduction [93, 94]. Postprogression strategies following pembrolizumab‐based therapy remain undefined, as subsequent dual ICI therapy is not recommended and evidence for PD‐1 inhibitor rechallenge or interclass switching is limited. In this context, enrolment in clinical trials, selective platinum‐based re‐challenge, and biomarker‐driven translational research constitute investigational approaches, emphasizing the ongoing need for evidence‐based sequencing strategies in PM.

According to the FLORA study, a multicenter retrospective Dutch study, 47% of patients with progressive disease after first‐line treatment with the combination of nivolumab and ipilimumab were eligible for second‐line therapy. The study reported a median OS of 8.2 months, a median PFS of 5.6 months, and an ORR of 37% for second‐line therapy with platinum derivatives and chemotherapy. Based on these data, second‐line chemotherapy could be a rational option for patients with PM previously treated with immunotherapy regimens [95].

Several studies [96, 97, 98, 99] suggest a potential correlation between the response to the first line of treatment and benefit from second‐line therapy. In particular, patients who had a longer duration of response to first‐line treatment demonstrated a significantly longer median OS (*p* = 0.039), with better PFS observed in those with a longer response [100].

In addition to PD‐1 and CTLA‐4, other immune system‐targeting strategies are being explored as promising new approaches [101]. VISTA (V‐domain Ig‐containing suppressor of T‐cell activation), a gene similar to PD‐L1, can suppress T‐cell activity when overexpressed on T lymphocytes. VISTA acts as both a ligand on antigen‐presenting cells and a receptor on T‐cells, with VSIG‐3 serving as its ligand [102]. LAG‐3, an inhibitory receptor on T‐cells, plays a role in T‐cell exhaustion and suppresses both T‐cell activation and cytokine secretion [103]. Although LAG‐3 is not typically expressed on mesothelial cells, high levels have been found in pleural effusions of mesothelioma patients. Targeting LAG‐3 is an active research area for mesothelioma treatment [104].

**FIGURE 5:**
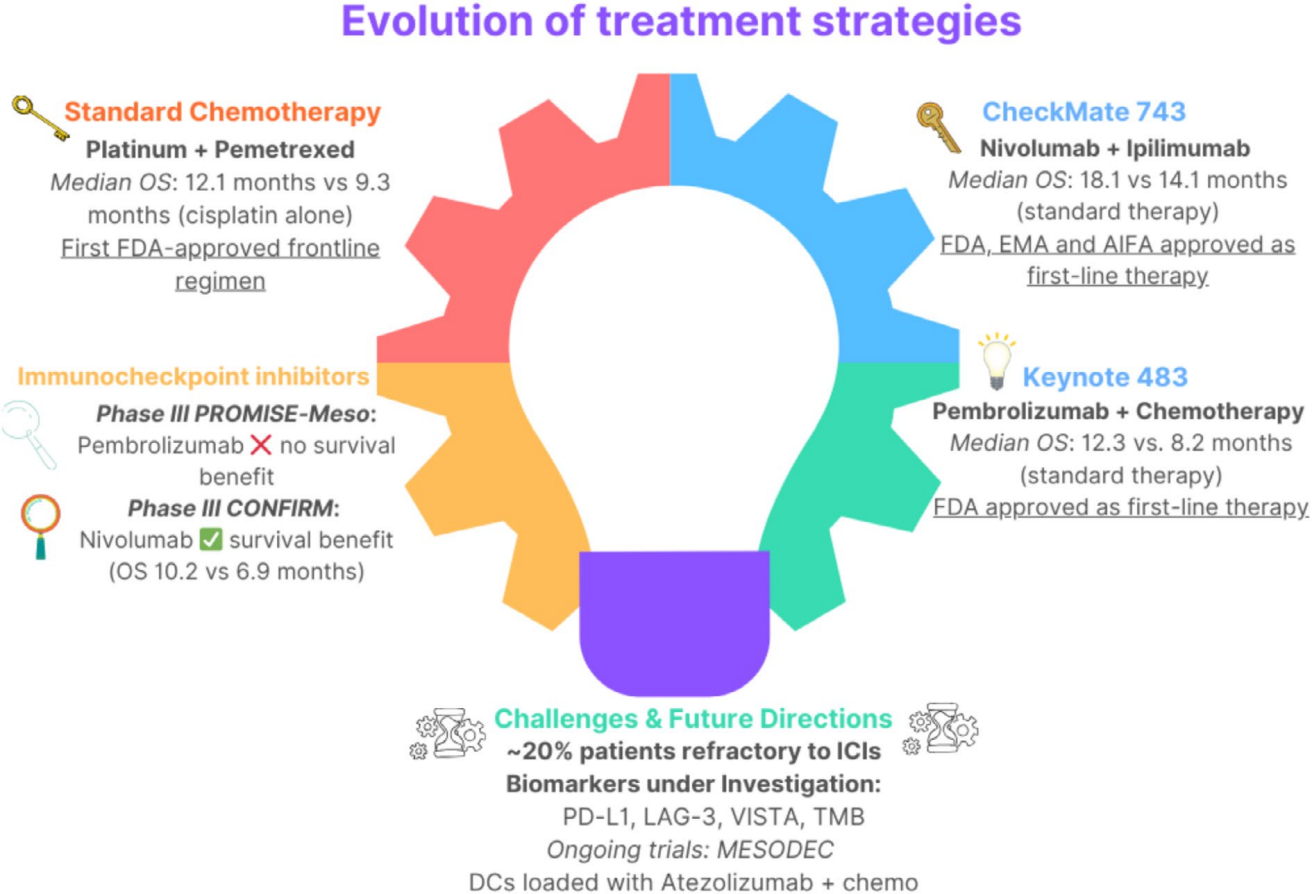
Evolution of treatment strategies for PM. The figure summarizes the evolution of treatment for PM with key clinical trials that have shaped the treatment landscape, from platinum‐pemetrexed chemotherapy (EMPHACIS) to immune checkpoint inhibitors (CheckMate 743 and Keynote 483) and ongoing research into novel targets such as VISTA, LAG‐3, and dendritic cell‐based therapies (MESODEC).

Nonetheless, in a multicenter Phase I/II trial (MESODEC study), the PD‐L1 inhibitor Atezolizumab and dendritic cells (DCs) filled with the PM‐associated tumor antigen WT1 may be integrated with the CT treatment based on platinum‐derived and pemetrexed for the treatment of epithelioid PM. DCs are potent activators of CD8+ T cells and could contribute to establishing a long‐lasting antitumor immunity and improve the patient's outcomes while WT1 is a potentially potent biomarker for PM because it is overexpressed in up to 99% of cases. The study is ongoing with the estimated primary completion date and study completion date set for October 2026 [105]. The summary of the treatment strategies for PM was represented in Figure 5.

## 2 Conclusion

PM is an aggressive cancer, typically identified in its later stages, with a poor prognosis. Asbestos exposure remains the primary risk factor leading to progressive inflammation, DNA damage, and tumorigenesis. The presence of unexplained pleural effusion and chest pain in patients with a history of asbestos exposure should raise concern for PM. Thoracoscopic biopsy is still the most reliable method for a definitive diagnosis, but the use of TUS‐guided percutaneous needle biopsy should be considered for diagnosing PM, especially in patients who are not candidates for thoracoscopy. Therapeutic strategies have evolved with the approval of nivolumab and ipilimumab, offering significant survival benefits, especially for nonepithelioid subtypes. New immune strategies targeting VISTA, LAG‐3, and dendritic cells represent promising areas of exploration for the future treatment of PM. Early detection, improved biomarker identification, and better understanding of the tumor microenvironment are crucial for enhancing patient outcomes.

## Author Contributions

**Raffaella Pagliaro:** conceptualization, formal analysis, investigation, data curation, writing – original draft preparation. **Beatrice Leonardi:** conceptualization, investigation, writing – original draft preparation. **Angela Schiattarella:** validation, investigation, writing – original draft preparation. **Grazia Bergameo:** validation, investigation, writing – original draft preparation. **Carmine Picone:** software, data curation. **Adolfo Gallipoli D'Errico:** validation, resources, data curation. **Filippo Scialò:** software, writing – review and editing, visualization, supervision. **Fabio Perrotta:** software, visualization, supervision. **Maria Luisa De Rimini:** methodology, resources. **Alfonso Fiorelli:** methodology, writing – review and editing, project administration. **Andrea Bianco:** methodology, writing – review and editing, project administration. All authors have read and agreed to the published version of the manuscript.

## Funding

The authors have nothing to report.

## Conflicts of Interest

The authors declare no conflicts of interest.

## Data Availability

Data sharing not applicable to this article as no datasets were generated or analysed during the current study.
